# DNA Repair and Signaling in Immune-Related Cancer Therapy

**DOI:** 10.3389/fmolb.2020.00205

**Published:** 2020-09-08

**Authors:** Sangeeta Kakoti, Hiro Sato, Siddhartha Laskar, Takaaki Yasuhara, Atsushi Shibata

**Affiliations:** ^1^Signal Transduction Program, Gunma University Initiative for Advanced Research (GIAR), Maebashi, Japan; ^2^Department of Radiation Oncology, Gunma University, Maebashi, Japan; ^3^Department of Radiation Oncology, Tata Memorial Centre, Mumbai, India; ^4^Laboratory of Molecular Radiology, Center for Disease Biology and Integrative Medicine, Graduate School of Medicine, The University of Tokyo, Tokyo, Japan; ^5^Massachusetts General Hospital Cancer Center, Harvard Medical School, Charlestown, MA, United States

**Keywords:** DNA repair, non-homologous end joining, homologous recombination, ataxia-telangiectasia-mutated, ataxia telangiectasia and Rad3-related, immune checkpoint inhibitor

## Abstract

Cancer therapy using immune checkpoint inhibitors (ICIs) is a promising clinical strategy for patients with multiple types of cancer. The expression of programmed cell death ligand-1 (PD-L1), an immune-suppressor ligand, in cancer cells is a factor that influences the efficacy of ICI therapy, particularly in the anti-programmed cell death protein-1 (PD-1)/PD-L1 antibody therapy. PD-L1 expression in cancer cells are associated with tumor mutation burden including microsatellite instability because the accumulation of mutations in the cancer genome can produce abnormal proteins via mutant mRNAs, resulting in neoantigen production and HLA-neoantigen complex presentation in cancer cells. HLA-neoantigen presentation promotes immune activity within tumor environment; therefore, known as hot tumor. Thus, as the fidelity of DNA repair affects the generation of genomic mutations, the status of DNA repair and signaling in cancer cells can be considered prior to ICI therapy. The Cancer Genome Atlas (TCGA) and The Cancer Immunome Atlas (TCIA) database analysis showed that tumor samples harboring mutations in any non-homologous end joining, homologous recombination, or DNA damage signaling genes exhibit high neoantigen levels. Alternatively, an urgent task is to understand how the DNA damage-associated cancer treatments change the status of immune activity in patients because multiple clinical trials on combination therapy are ongoing. Recent studies demonstrated that multiple pathways regulate PD-L1 expression in cancer cells. Here, we summarize the regulation of the immune response to ICI therapy, including PD-L1 expression, and also discuss the potential strategies to improve the efficacy of ICI therapy for poor responders from the viewpoint of DNA damage response before or after DNA damage-associated cancer treatment.

## Introduction

The clinical application of immune checkpoint inhibitors (ICIs), such as anti-programmed cell death protein-1 (PD-1)/programmed cell death ligand-1 (PD-L1) and anti-cytotoxic T lymphocyte-associated protein 4 (CTLA-4) antibodies, has improved the clinical outcome of patients with various malignancies. Practically, anti-PD-1/PD-L1 antibodies have been widely used at the clinical level. However, the development of a new clinical strategy remains essential because only a limited number of patients respond to anti-PD-1/PD-L1 therapy (Sharma et al., 2017a). For the selection of responders or the improvement of the therapeutic efficacy in non-responders, recent studies have actively sought to identify an optimal biomarker for the prediction of treatment response to ICI therapy (Galon and Bruni, 2019). PD-L1, a ligand that is expressed on cancer cell surface, binds to PD-1 that is a T-cell surface receptor. The binding stimulates a signal transduction within T cell, which subsequently suppresses the immune activity and proliferation of T cells (Iwai et al., 2002). Thus, the presence of PD-L1 on cancer cell surface influences the overall immune activity in tumor. The mechanistic basis for ICI therapy is that the PD-L1 expression in cells within tumor environments is also considered to be essential because anti-PD-1/PD-L1 antibodies target and inhibit the interaction between PD-1 and PD-L1, restoring immune activity in the tumor environment. As another biomarker for anti-PD-1/PD-L1 therapy, microsatellite instability (MSI) is widely used. The accumulation of mutations in tumor genome, also known as tumor mutation burden (TMB), is related to the formation of abnormal proteins, because mutations in genes at transcriptionally active loci produce mutant mRNAs that subsequently form HLA-neoantigen complex following the generation of peptides by proteasome-dependent degradation of abnormal proteins (Schumacher and Schreiber, 2015). The presentation of HLA-neoantigen on the surface of cancer cells promotes immune activity and transforms cold tumors into hot tumors. Despite the upregulation of the HLA-neoantigen-dependent immune activation, it is still not sufficient to overcome the progression of cancers under the immune-suppressive environment. Such hot tumors are considered to be sensitive to ICIs. The alleviation of immune suppression by ICIs will switch on the immune activity. Thus, the status of DNA repair in cancer cells might be important because the fidelity of repair influences the amount of TMB including MSI.

To date, studies have shown that the PD-L1 expression in tumors is influenced by DNA repair and signaling via multiple pathways. Prior to cancer treatment, endogenous DNA damage in tumors could be persistently generated due to oxidative stress or abnormal cell cycling. Under such circumstances, DNA damage responses (DDR) may upregulate immunological signaling. However, the immune activity under the situation without additional exogenous DNA damage, e.g., prior to radiotherapy (RT)/chemotherapy, is not completely able to overcome cancers. This situation will be likely in case of low TMB/MSI tumors. In contrast, recent studies have shown that multiple immunological responses including the release of interferons (IFNs, immune positive response) and PD-L1 upregulation (immune negative response) are induced after DNA damage-associated cancer treatments, such as RT and chemotherapy (McLaughlin et al., 2020). After RT/chemotherapy, both immune positive (HLA-neoantigen) and negative responses (PD-L1 upregulation) are activated, and the negative responses can be cancelled by ICIs. Thus, the introduction of DNA damage can be a trigger to transform tumors from cold to hot, irrespective of the TMB/MSI status. Here, we review the molecular linkage between immune-response and DDR, including the latest findings in the field.

## Mechanism of Immune-Mediated Cancer Cell Killing

Tumor cells often harbor many “passenger” mutations, in addition to the carcinogenic “driver mutations” (McFarland et al., 2017). Among passenger mutations, non-synonymous mutations produce mRNAs containing mutations that alter the amino acid sequence of proteins, and these abnormal proteins get proteolytically degraded into short peptides. The endogenous Major Histocompatibility Complex class I (MHC-I, HLA in human) recognizes these peptides as neoantigens, which are then transferred to and presented at the tumor cell surface. This HLA-neoantigen complex then activates cytotoxic T cells by binding to the T-cell receptor (TCR) either directly or via the cross-presentation by professional antigen-presenting cells (APCs), such as dendritic cells (Garbi and Kreutzberg, 2012). Activated T cells subsequently eliminate tumor cells by a series of cytolytic events that mainly involve cytokine release (e.g., perforin and granzyme) (Martinez-Lostao et al., 2015). Thus, non-synonymous mutations in tumor cells eventually upregulate the immune activity in the tumor environment and kill cancer cells via the immune system. Recently, a phase I clinical trial in which a neoantigen vaccine was administered to glioblastoma patients showed that CD4^+^ and CD8^+^ T cells are activated in a neoantigen-dependent manner (Keskin et al., 2019). This result supports the notion that neoantigen is a key factor in immune-dependent cancer cell killing effect.

**FIGURE 1:**
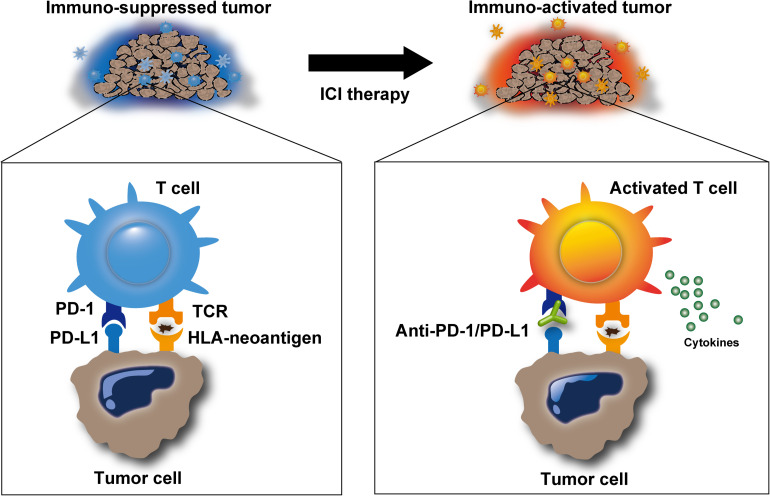
Release of immune suppression by immune checkpoint inhibitors. Immune checkpoint inhibitors, such as anti-programmed cell death protein-1 (PD-1)/programmed cell death ligand-1 (PD-L1) antibodies, inhibit immunosuppression in the tumor microenvironment and promote T-cell activation. Activated T cells can release cytotoxic molecules, including perforin, granzyme, and IFNγ, to eliminate target cells.

The overt manifestation of clinical malignant tumors is usually the result of “escape” from the immune-mediated “elimination” of tumor cells described above (the steps involving elimination, equilibrium, and escape are collectively termed as “Cancer Immunoediting”) (Dunn et al., 2002). Broadly, this may occur due to defects or dysfunction in any of the steps involved in elimination, such as the lack of immunogenic neoantigens on tumors, dysfunctional APCs that cannot effectively prime the neoantigens, dysfunctional cytotoxic T cells that are unable to become activated or secrete cytokines, and impaired activity of cytokines, including their receptor or ligand inactivity (Figure 1, left) (Matsushita et al., 2012; Schumacher and Schreiber, 2015; Sharma et al., 2017a). Importantly, these immune escape mechanisms also promote the resistance for immunotherapy (O’Donnell et al., 2019). Among the various mechanisms involved in the immunological escape of tumor cells, activated immune checkpoint pathways, including the PD-1/PD-L1 and CTLA-4 axis, are the central suppressors amenable to therapeutic inhibition (Figure 1, left). Specifically, PD-L1 protein expressed on tumor cells binds to PD-1 receptors on cytotoxic T cells, preventing T cells from being activated by the HLA-neoantigen complex (immune exhaustion) (Thommen and Schumacher, 2018). CTLA-4 is a member of the CD28 family of receptors that is constitutively expressed on regulatory T (Treg) cells, a type of T cell, which secrete inhibitory cytokines, such as interleukin (IL)-10, IL-35, and transforming growth factor (TGF)-β, or directly contact effector T cells, subsequently suppressing T-cell responses.

The history of the use of immunotherapy to overcome the immune-mediated escape of tumor cells began with a stimulation of host immunity. The administration of attenuated bacteria (Coley’s toxin) alone in inoperable sarcomas and carcinomas showed unprecedented results (5-year overall survival rates up to 79 and 73%, respectively) in the early 1900s (Hoption Cann et al., 2003). The *ex vivo* activation of cytotoxic T cells, followed by infusion back into the patients’ body (a procedure termed adoptive T-cell therapy) also showed promising results, especially in the treatment of advanced melanoma (Rosenberg et al., 2011). Alternatively, to date, ICIs, which release the patients’ intrinsic immune response from the suppressed state, have provided a significant leap in the evolution of anticancer immunotherapy (Figure 1). ICI treatment exerts antitumor activity by restoring the intrinsic immune response, as described above, in patients who are suppressed by these immune checkpoints. Anti-PD-1/PD-L1 antibodies aim to block the undesired “T-cell-exhausting” communication between PD-L1 on tumor cells and its receptor PD-1 on cytotoxic T cells. Based on this mechanism, PD-1/PD-L1 blockade has resulted in considerable improvements in the outcomes of patients with advanced malignancies across a wide range of tumor sites (Brahmer et al., 2015; Sharma et al., 2017b). Similarly, anti-CTLA-4 antibody therapy has shown impressive results in metastatic melanoma patients (Margolin et al., 2012). CTLA-4 is expressed on activated T cells, whereas CD80/86 is expressed on APCs, including tumor cells. Anti-CTLA-4 antibodies block the interaction between CTLA-4 and CD80/86, resulting in immune activity restoration. Anti-PD-1/PD-L1 blockade primarily affects the immune activity in tumor environment, whereas anti-CTLA-4 therapy restores the immune activity throughout the lymph tissues. Thus, each therapy differently targets the area of patient body; therefore, synergistic effect can be obtained using a combination of both inhibitors. A combination of inhibitors targeting both of them has the potential to improve patient outcomes compared with either one of them alone (Larkin et al., 2015; Hellmann et al., 2018; Overman et al., 2018).

Although such elegant mechanisms underlie the basis of ICI therapy, only 5% of patients are categorized as high responders, indicating that approximately 95% patients may not be effectively cured. Thus, present efforts in the field of immunotherapy are focused on identifying suitable biomarkers to predict candidates who benefit from ICI treatment. High TMB is thought to be associated with the generation of increased immunogenic neoantigens and better response rates to ICIs (Rizvi et al., 2015). In fact, a high MSI score, which is induced by defects in mismatch repair, is highly related to ICI efficacy. Therefore, MSI is currently used as one of the most reliable predictive biomarkers for ICI therapy (Le et al., 2017; Marabelle et al., 2020). Several studies have shown that base excision repair (BER) defects, chromatin remodeling, and DNA replication also contribute to MSI (Garcia-Sanz et al., 2017; Matsuno et al., 2019) (see further discussion below). In addition to the TMB/MSI/neoantigen axis, high PD-L1 expression level on the tumor cell surface is also found to be a favorable predictive biomarker (Topalian et al., 2016). In the next section, we discuss how DDRs associate with neoantigen production.

## Potential Regulation of Neoantigen Production in DNA Damage Response Defective Cancers

**FIGURE 2:**
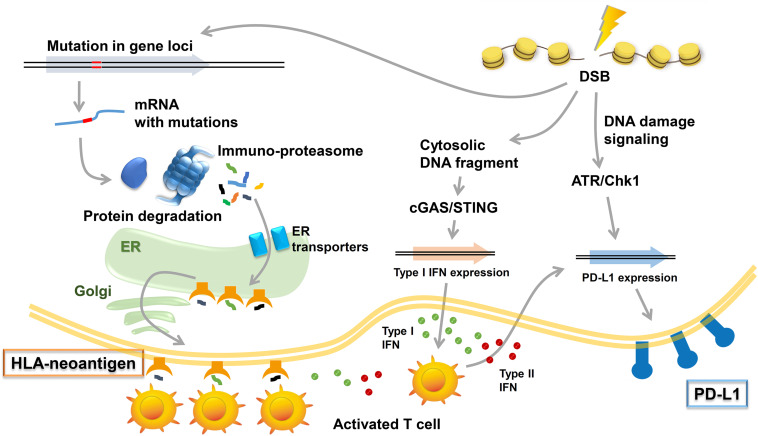
Immune responses induced by DNA damage response (DDR). Radiotherapy (RT)/chemotherapy change the immune microenvironment to be advantageous in combination with immune checkpoint inhibitors. Three types of immune responses are included in these responses: (1) neoantigen production induced by mutations in gene loci and the induction of HLA class I (HLA)-neoantigen complex expression, (2) PD-L1 upregulation via DNA damage signals, and (3) the accumulation of cytoplasmic DNA and activation of the cGAS/STING pathway.

DNA damage responses (both repair and signaling) are vital to ensure genome stability; thus, DDR defective status is often associated with somatic mutations (Jackson and Bartek, 2009) that frequently lead to frameshift errors and abnormal protein synthesis following transcription and translation. Peptides, presented as HLA-neoantigen, are produced by immunoproteasomes. Immunogenic neoantigens are transported to the endoplasmic reticulum (ER), and the HLA-neoantigen complex is formed followed by its transfer to the cell surface (Figure 2). The presented HLA-neoantigen interacts with TCR on T cells, and this interaction promotes T-cell activity enhancing the tumor cell killing effect (Schumacher et al., 2019). In contrast to the positive effect by HLA-neoantigen dependent immune stimulation, the release of IFNs from the activated T cells promotes the expression of PD-L1 in tumor environment (Garcia-Diaz et al., 2017).

**FIGURE 3:**
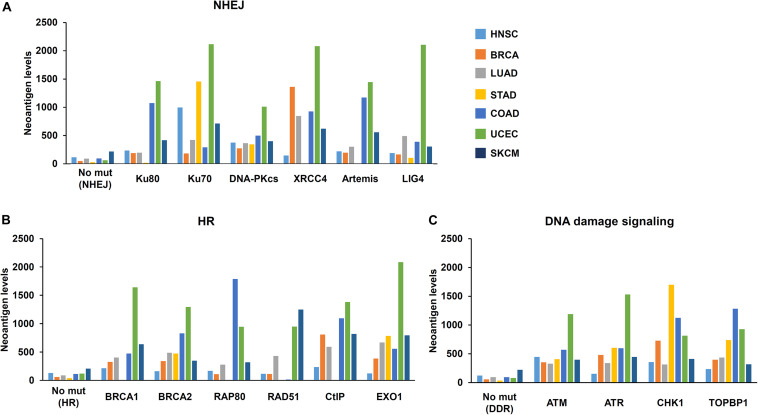
Mutations in DNA repair pathways and neoantigen production level. The correlation between mutations in each DNA repair pathway and the level of neoantigen production was analyzed. Mutation statuses provided by TCGA project were downloaded from the Genomic Data Commons Data Portal. The neoantigen data were obtained from The Cancer Immunome Atlas (TCIA)^1^. Levels of neoantigen in samples harboring mutations, including indels and point mutations in NHEJ, HR, or DNA damage signaling genes, are shown. Neoantigen levels in the y axes represent the average number of neoantigens per tumor sample in each group. The number of samples (*N*) in this study is listed in Table 1. **(A)** Genes involved in non-homologous end joining (NHEJ). **(B)** Genes involved in homologous recombination (HR). **(C)** Genes involved in the DNA damage signaling. HNSC, head and neck squamous cell carcinoma; BRCA, breast invasive carcinoma; LUAD, lung adenocarcinoma; STAD, stomach adenocarcinoma; COAD, colon adenocarcinoma; UCEC, uterine corpus endometrial carcinoma; SKCM, skin cutaneous melanoma.

**FIGURE 4:**
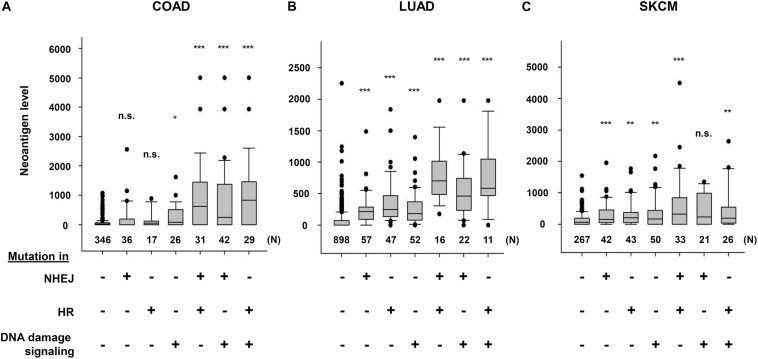
Multiple DNA repair pathway mutations and neoantigen production level. The correlation between mutations in multiple DNA repair pathways and neoantigen production levels using The Cancer Genome Atlas (TCGA) and The Cancer Immunome Atlas (TCIA) dataset. The distribution of the number of neoantigens in each group is shown by box plots. **(A)** Colon adenocarcinoma (COAD). **(B)** Lung adenocarcinoma (LUAD). **(C)** Skin cutaneous melanoma (SKCM). The analysis of neoantigen levels are described in the legend of Figure 3. Box plots were created using SigmaPlot 12.0. Statistical analysis was performed by Student’s two-tailed *t*-test by SigmaPlot. *p < 0.05, **p < 0.01, ***p < 0.001.

The Cancer Genome Atlas (TCGA) and The Cancer Immunome Atlas (TCIA),a publicly accessible genomic dataset of clinical specimens, are a useful tool to analyze gene expressions, TMB and neoantigen production predicted by genomic mutations (Hutter and Zenklusen, 2018). Defect of mismatch repair (MMR) activity is considered to be a major cause of generation of TMB and neoantigen production in cancer cells. Mutations in MMR genes are associated with significantly increased neoantigen production and distinct immunological characteristics in lung squamous cell carcinoma (Chae et al., 2019). We previously reported that tumors with mutations in any of the double-strand break (DSB) repair genes show enhanced PD-L1 expression (Sato et al., 2017). Further, tumors with mutations in any of the BER genes exhibit statistically significant increases in neoantigen production and PD-L1 expression (Permata et al., 2019). Other dataset analyses have shown that high-grade serous ovarian cancer patients with homologous recombination (HR) deficiency have a higher neoantigen load and an increase in PD-1 and PD-L1 expression compared with HR proficient patients (Strickland et al., 2016). Another database study reported that patients with simultaneous mutations in HR and MMR, or HR and BER are associated with increased TMB and neoantigen production (Wang et al., 2018). Importantly, patients with high TMB/neoantigen in HR-MMR- or HR-BER-defective groups show a favorable clinical benefit from ICI therapy, suggesting that these DNA repair factors also can be supportive biomarkers for ICI therapy. Thus, DDR-induced neoantigen production, and subsequent immune-stimulation and PD-L1 expression, are being clarified. In this section, we present our analysis of neoantigen production in relation to the status of DDR, which is categorized by non-homologous end joining (NHEJ), HR, or DNA damage signaling by using TCGA and TCIA. To investigate the impact of NHEJ, HR, or DNA damage signaling on neoantigen production derived from genomic mutations, the neoantigen levels in patients with various tumor sites were analyzed (Figures 3A–C; representative genes of NHEJ, HR, and DNA damage signaling are summarized in Table 1). Importantly, we found increased levels of neoantigen production in tumors with mutations in either NHEJ, HR, or DNA damage signaling pathways. Neoantigen production increase was particularly evident in uterine corpus endometrial carcinoma (UCEC), stomach adenocarcinoma (STAD), and colon adenocarcinoma (COAD) across multiple genes (Figures 3A–C). We further analyzed the impact of the mutations in combination with NHEJ, HR, and DNA damage signaling on neoantigen production (Figure 4). Notably, a combination of mutations in different pathways (NHEJ + HR, NHEJ + DNA damage signaling, and HR + DNA damage signaling) showed higher levels of neoantigen production compared with mutations in one of the pathways (Figures 4A–C), which might be attributed to a compromised availability of alternate repair pathways, resulting in a higher frequency of neoantigen production when genes of more than one pathway are mutated. In combination with DNA damage signaling mutant, the NHEJ or HR mutant may cause further mutations in genes producing neoantigen under the deficiency of cell-cycle checkpoint, which arrests cell cycle at the G1/S or G2/M boundary (see below about cell-cycle checkpoint arrest). The produced neoantigen should be bound to HLA to form the HLA-neoantigen complex, even if not all, and neoantigen production increase under DDR deficiency will activate T cells, leading to the release of immune cytokines and enhanced total immune activity in the tumor environment. However, this signaling also upregulates PD-L1 expression, and ICI treatment will be effective in this situation. The result of the database analysis is supported by a recent study by Cheng’s group, which reported that the analysis of tumor samples from patients with non-small cell lung cancer treated with anti-PD-1/PD-L1 antibody using next-generation sequencing revealed that patients with DDR mutation (the most commonly mutated DDRs were *ATM*, *ATR*, *BRCA2*, *POLQ*, and *RAD50* in the analysis) exhibited high TMB. The patients harboring mutations in DDR genes had longer progression-free survival and overall survival under an ICI treatment (Ricciuti et al., 2020).

**TABLE 1:** Analysis of NHEJ, HR, and DDR pathway mutation and neoantigen production by TCGA dataset.

Repair pathway	Analyzed Gene	Sample number						
		HNSC	BRCA	LUAD	STAD	COAD	UCEC	SKCM
NHEJ	No mutations	451	1042	999	315	394	514	380
	Ku80	6	3	12	6	17	28	10
	Ku70	4	8	8	7	14	24	13
	DNA-PKcs	31	45	51	43	55	103	63
	XRCC4	4	6	1	4	9	19	5
	Artemis	5	8	7	8	6	26	6
	LIG4	9	8	17	7	13	36	13
HR	No mutations	455	1034	1020	327	424	537	376
	BRCA1	11	24	20	7	11	37	26
	BRCA2	20	31	27	24	32	79	39
	RAP80	7	5	4	6	8	28	22
	RAD51	2	4	1	2	2	17	2
	CtIP	5	10	6	6	8	26	20
	EXO1	3	12	14	13	13	25	14
DDR	No mutations	455	1051	1010	322	407	514	381
	ATM	14	29	37	31	49	91	46
	ATR	26	22	23	22	20	66	48
	CHK1	1	5	7	6	7	17	5
	TOPBP1	10	13	12	14	13	44	16

*HNSC, head and neck squamous cell carcinoma; BRCA, breast invasive carcinoma; LUAD, lung adenocarcinoma; STAD, stomach adenocarcinoma; COAD, colon adenocarcinoma; UCEC, uterine corpus endometrial carcinoma; SKCM, skin cutaneous melanoma.*

As described above, neoantigens, which are presented by HLA on the cell surface, are derived from peptides following the degradation of abnormal proteins, although not all peptides are recognized by HLA. Thus, the generation of mutations within the coding regions of gene loci is the origin of neoantigen production. MMR deficiency and DNA replication errors cause genome-wide mutations, including mutations in gene loci. A defect in other DNA repair mechanisms, such as NHEJ, HR, BER, or nucleotide excision repair, can also lead to the generation of mutations when exogenous DNA damage is induced at transcriptionally active loci. Defects in factors required for transcription-associated DNA repair at gene loci may further increase the mutation rate in the neoantigen production. Taken together, DDR status is a potential prognostic biomarker in ICI therapy.

## Regulation of PD-L1 Expression in the Context of DNA Damage Response

**FIGURE 5:**
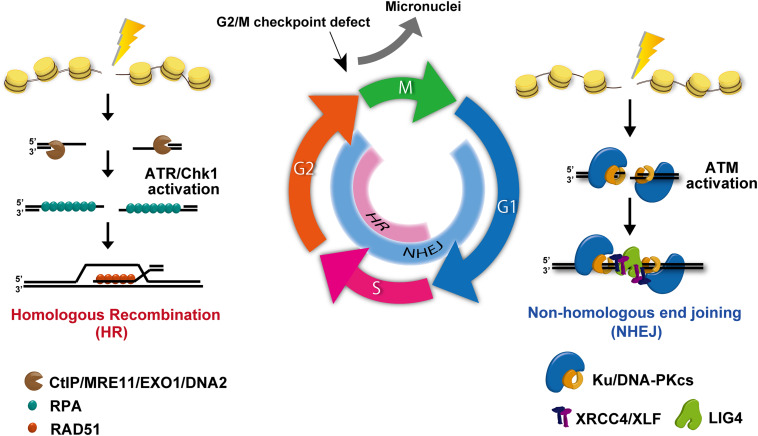
DNA double-strand break (DSB) repair and signal activation. Non-homologous end joining (NHEJ) functions throughout all cell-cycle phases except mitosis. Homologous recombination (HR) becomes active and repairs DSBs only in the S/G2 phase. During repair, ataxia-telangiectasia-mutated (ATM) is activated at un-resected DSB ends, i.e., at the breaks undergoing NHEJ. In addition, ataxia telangiectasia and Rad3-related (ATR) is effectively activated at ssDNA following DSB end resection. Because DSB ends are resected during HR, ATR activation occurs in the S/G2 phase.

RT and DNA damage-associated chemotherapy, such as platinum-based drugs, are widely used anticancer treatments, which act primarily by generating lethal DNA damage leading to cell death and restricted cell proliferation. DSBs are the most lethal type of DNA damage. The failure of DSB repair causes lethal mutations or cell death. In human cells, DSBs are repaired by NHEJ or HR pathways (Shibata and Jeggo, 2014). In addition to the two major pathways, alternative end joining, which is a deleterious repair pathway due to the use of a microhomology sequence for the rejoining following DSB end resection, is also used in cancer cells (Trenner and Sartori, 2019). This pathway is activated when the expression of a core NHEJ component, such as Ku70/80, is reduced. The use of NHEJ and HR is regulated by the cell cycle. For example, NHEJ functions throughout cell-cycle phases, whereas HR repairs DSBs during the S/G2 phase (Figure 5). Therefore, in proliferating cancer cells, drugs targeting and inhibiting HR in the S/G2 phase have been developed for cancer treatment (Bai et al., 2017). During repair, cells need to arrest cycling until DSBs are repaired. This cell-cycle checkpoint arrest is activated by a DNA damage signaling pathway, which is initiated at the site of DSBs. Ataxia-telangiectasia-mutated (ATM) and ataxia telangiectasia and Rad3-related (ATR) are central regulators in cell-cycle checkpoint arrest. These two kinases are distinctly activated in each cell-cycle phase. Because ATM is preferentially activated at the ends of DSBs during NHEJ, ATM greatly contributes to G1/S checkpoint arrest via the checkpoint kinase 2 (Chk2)/p53 pathway. In contrast, ATR is activated during HR and contributes to G2/M checkpoint arrest. In general, G1/S checkpoint arrest is downregulated due to the absence of the p53 pathway in cancer cells. Therefore, DNA damaged cancer cells often accumulate in the G2 phase by ATR/Chk1-dependent checkpoint arrest.

Several reports demonstrate that PD-L1 expression is upregulated by RT or chemotherapy (Lim et al., 2016, 2017; Kelly et al., 2018; Yoneda et al., 2019). Recently, we reported that DSBs upregulate PD-L1 expression in a transcription-dependent manner via the STAT-IRF1 pathway (Figure 2; Sato et al., 2017). In this scenario, ATM, ATR, and Chk1 were found to be important for DSB-induced PD-L1 upregulation. More importantly, the depletion of DNA repair proteins, including Ku80 and breast invasive carcinoma (BRCA) 2, was found to enhance PD-L1 upregulation after DSBs, suggesting that patients with mutations of these proteins may highly express PD-L1 and thus benefit more from consolidative PD-1/PD-L1 blockade after RT or chemotherapy. Similar to the response to DSBs, oxidative stress also upregulates PD-L1 expression via the ATR/Chk1/STAT/IRF1 pathway, which is further enhanced by the depletion of BER factors (Permata et al., 2019). Furthermore, ATR inhibition downregulates PD-L1 expression in tumor cells by destabilizing PD-L1 in a proteasome-dependent manner and has resulted in enhanced immune cell killing (Sun et al., 2018). In addition, ATR inhibition attenuates irradiation-induced PD-L1 upregulation and decreases the number of tumor-infiltrating Tregs in mouse models (Vendetti et al., 2018). In contrast to these responses in cancer cells, DNA damage-induced upregulation of PD-L1 was not observed in normal human dermal fibroblasts (Hagiwara et al., 2018).

Another mechanism that regulates PD-L1 expression in response to DNA damage is the cyclic-GMP-AMP synthase (cGAS)/stimulator of IFN genes (STING) pathway. In general, cancer cells can activate G2/M checkpoint arrest, whereas G1/S checkpoint arrest is frequently lost due to the lack of p53 pathway. Despite the activation of G2/M checkpoint signaling, G2/M checkpoint machinery in human cells is not perfectly able to sustain checkpoint arrest, even if all DSBs in G2 cells have not been repaired (Deckbar et al., 2007). Indeed, premature checkpoint release can occur even in normal human cells (Deckbar et al., 2007). Once G2 cells with DSBs progress into mitosis, these cells can generate micronuclei. Additionally, micronuclei can be generated during mitosis if chromosomal aberrations such as dicentric or acentric are formed by misrepair (Figure 5). The loosely bound DNA inside micronuclei with ruptured nuclear envelopes recruit the DNA sensor protein cGAS, which then transduces the signaling toward STING (Ablasser et al., 2013; Harding et al., 2017; Mackenzie et al., 2017). cGAS/STING pathway activation eventually induces the mRNA expression of type-I IFN via an IRF3/NFκB-dependent transcriptional pathway. The produced type-I IFNs is released and incorporated into cells via IFN receptors, subsequently upregulating PD-L1 in cancer cells. Thus, cGAS/STING pathway is considered to contribute to PD-L1 regulation as well as other cytokines. Supporting this notion, DNA damage-induced activation of the cGAS-STING-type-I pathway is also implicated in the induction of PD-L1 expression in mouse models (Grabosch et al., 2019). Therefore, micronuclei and the cGAS/STING cascade are involved in PD-L1 upregulation when cells are released from G2/M checkpoint arrest after DNA damage. Importantly, in addition to cell-cycle checkpoint factors, the status of DNA repair factors also influences the generation of micronuclei. DNA repair-deficient breast cancer cells derived from patient samples were found to contain increased cytosolic DNA (specific to S phase DNA damage), followed by constitutive PD-L1 expression via the cGAS-STING-type-I IFN pathway (Parkes et al., 2017). Specifically, NHEJ deficiency causes a reduction in the number of irradiation-induced micronuclei due to prolonged G2/M checkpoint arrest (Harding et al., 2017), whereas depleting HR proteins have an opposite effect, suggesting that HR deficiency causes mitotic abnormalities via DNA replication (Feng and Jasin, 2017). In fact, although HR is known to repair DSBs in the S/G2 phase, the majority of DSB repair in G2 phase is carried out by NHEJ (Figure 5; Shibata, 2017). Therefore, the status of NHEJ activity also influences micronuclei formation.

PARP inhibitors, which cause DNA replication-associated DNA damage, induces PD-L1 upregulation (Jiao et al., 2017). Another study shows that this upregulation is dependent on the cGAS/STING pathway and the activation of cGAS/STING pathway is mediated via cytoplasmic DNA (Shen et al., 2019). Recent early clinical trials reported the result of a combination of PARP inhibitor and ICIs (Friedlander et al., 2019; Zimmer et al., 2019; Lampert et al., 2020). These are promising approaches; however, further investigation is required to clarify the clinical benefit of this combination therapy. The contribution of DNA fragment generated at stalled replication fork has been also reported (Coquel et al., 2018). In normal cells, MRE11 exonuclease in association with phosphorylated SAMHD1 digests nascent DNA strands at the stalled DNA replication fork, whose fragment is not recognized by the cGAS/STING pathway. However, in the absence of SAMHD1, MRE11 endonuclease, but not exonuclease, creates a nick at the nascent DNA strand, generating a larger DNA fragment, which is recognized by cGAS following transport into the cytosol and this promotes STING-dependent IFN release. Other DNA repair-/replication-related factors that activate the cGAS/STING pathway are cytosolic RNA:DNA hybrids (Mankan et al., 2014) and telomere erosion (Chen et al., 2017). As an alternative pathway, ATM activates STING in a cGAS-independent manner (Dunphy et al., 2018). Thus, the status of DNA repair and signaling is also an important factor influencing immune activities, indicating that it has the potential to be a predictive biomarker for guiding ICI therapy, especially in combination with DNA damage-dependent cancer treatments. As another micronuclei-independent pathway, cGAS/STING pathway is also controlled by the levels of TREX1, a cytosolic nuclease (Stetson et al., 2008; Vanpouille-Box et al., 2017). The fractionated radiation, e.g., 3 × 8 Gy, does not upregulate TREX1; therefore, the cytosolic DNA fragments generated in response to radiation are able to activate cGAS/STING pathway. On the other hand, >20 Gy per fraction induces the expression of TREX1, which cleans up the desired immunogenic cytosolic DNA fragments. Therefore, a high dose irradiation, >20 Gy, does not effectively activate cGAS/STING pathway due to TREX1 upregulation (Vanpouille-Box et al., 2017). When >20 Gy are used in a clinical setting, the efficacy might be improved by TREX1 inhibition to maximize the irradiation-induced immunogenicity (Yamazaki and Galluzzi, 2017). The studies above suggest that the introduction of DNA damage by cancer therapy is involved in the activation of the immune response. Alternatively, Sen et al. (2019) showed that Chk1 inhibition activates the STING-TBK1-IRF3 pathway, increases PD-L1 expression, and, importantly, also augments cytotoxic T-cell infiltration. Another report showed that ATM deficiency increases the release of type-I IFN in a TBK1- and SRC-dependent but cGAS/STING-independent manner. Subsequently, the release of IFN leads to PD-L1 upregulation (Zhang et al., 2019).

Thus, ATM-ATR-Chk1-dependent DNA damage signaling within damaged tumor cells is involved in regulating PD-L1 expression in response to DNA damage. In parallel, micronuclei formation due to defects in repair and/or G2/M checkpoint arrest and DNA fragment formation activate the cGAS/STING pathway, releasing IFNs within the tumor environment, and its signaling upregulates PD-L1 expression via paracrine and autocrine pathways.

## Perspective for Precision Medicine for Immune Checkpoint Therapy in Combination With RT/Chemotherapy by Targeting DNA Repair and DNA Damage Signaling

In summary, evidence suggests that DNA repair and signaling are involved in the regulation of HLA-neoantigen presentation and PD-L1 expression. Therefore, the application of anti-PD-1/PD-L1 antibodies will be effective, particularly in combination with DNA damage-associated cancer treatments, such as RT and DNA-damaging chemotherapy. The mechanisms involved in the activation of the immune response after DNA damage include (1) HLA-neoantigen presentation to TCRs, (2) the ATM/ATR/Chk1 signal cascade, and (3) micronucleus/DNA fragment-dependent cGAS/STING activation. The signaling from cGAS/STING and HLA-neoantigens leads to both immune-activating and immune-suppressing responses. Under normal conditions without exogenous DNA damage (i.e., without cancer treatment), the deficiency of DDR may not sufficiently activate immune signaling. Thus, the balance of immune activity might still be toward immune suppression without additional stimuli although the presence of excessive mutations in genome may upregulate HLA-neoantigen pathway. In contrast, after DNA damage-associated cancer treatment, the immune environment may be changed from “cold tumors” to “hot tumors,” irrespective of the presence of mutations in tumors, enabling the modification of the overall immune status, which is sensitive to ICI therapy. Recent preclinical studies have shown that treatments targeting DDR alone or in combination with RT have immunostimulatory potential through micronuclei formation and type-I IFN downstream production (McLaughlin et al., 2020). Thus, DNA damage can be a powerful stimulus for immune activation. Despite the upregulation of PD-L1 expression following DNA damage, the immune-suppressive effect can be effectively inhibited by ICI therapy. Regarding the selection of combination therapy, RT might have an advantage in terms of the protection of immune cells in patients. A recent study elegantly demonstrated that a large proportion of T cells survive clinically relevant doses of radiation using longitudinal imaging of mice (Arina et al., 2019). Importantly, CD8 + tumor-infiltrating lymphocytes were recruited into the irradiated field after RT from the non-irradiated area. Further, the survived T cells after RT maintained cancer cell killing activities, such as the mobility and production of IFNs, even though T cells are generally radiosensitive.

To date, several studies have shown that such a combination exhibits a synergistic effect at the preclinical level. However, cancer is highly diverse; thus, it is essential to select the optimal approach for each patient based on the concept of precision medicine because some tumors may not express a key factor in a particular signaling pathway. For example, cGAS/STING is downregulated in some tumors. Regarding biomarkers for combination therapy, DDR status may be important for patients who receive DNA damage-associated cancer therapy. However, the timing of the balance of immune responses can be changed at any moment after RT and chemotherapy; thus, a biomarker may be appropriately selected based on the timing of ICI treatment after/during RT and chemotherapy. In the era of precision medicine, the elucidation of the mechanisms underlying the immune response in the context of DDR status will be important for considering the next generation of cancer therapy.

## Data Availability Statement

All datasets presented in this study are included in the article/supplementary material.

## Author Contributions

SK, HS, SL, and AS wrote the manuscript. TY developed the system of the TCGA and TCIA analysis. SK performed the TCGA analysis. AS designed the figures and HS made the table. All authors read and approved the final manuscript.

## Conflict of Interest

The authors declare that the research was conducted in the absence of any commercial or financial relationships that could be construed as a potential conflict of interest.

## Footnotes

1 http://tcia.at/
